# Association between Cataract Surgery and Age-Related Macular Degeneration: A Systematic Review and Meta-Analysis

**DOI:** 10.1155/2022/6780901

**Published:** 2022-05-05

**Authors:** Lihong Yang, Hongxun Li, Xinheng Zhao, Ye Pan

**Affiliations:** ^1^Tianjin Key Lab of Ophthalmology and Visual Science, Tianjin Eye Hospital, Tianjin, China; ^2^Clinical College of Ophthalmology, Tianjin Medical University, Tianjin, China

## Abstract

**Purpose:**

We performed a systematic review and meta-analysis to evaluate the association between cataract surgery and the development and progression of AMD.

**Methods:**

This meta-analysis was registered at PROSPERO (CRD42017077962). We conducted a systematic literature search in August 2020 in Embase and PubMed and included cohort studies, case-control studies, or randomized controlled trials (RCTs) if they examined the association between cataract surgery and AMD. Odds ratio (OR) was used as a measure of the association with a random effect model. The analysis was further stratified by factors that could affect the outcomes.

**Results:**

15 studies were included in this study. In the overall analysis, cataract surgery was significantly associated with the incidence of late AMD (OR, 1.80; 95% CI, 1.26–2.56; *P* = 0.001), particularly geographic atrophy (OR, 3.20; 95% CI, 1.90–5.39; *P* ≤ 0.001). No significant associations were observed between cataract surgery and the incidence of early AMD. Subgroup analysis showed that the OR for incidence of early and late AMD was significantly higher for cataract surgery performed more than 5 years compared with less than 5 years. We also found an increased risk of progression of AMD after cataract surgery performed more than 5 years (OR, 1.97; 95% CI, 1.29–3.01; *P* = 0.002).

**Conclusions:**

Our results suggest that cataract surgery may be associated with an increased risk of late AMD development and AMD progression. In addition, increasing the follow-up time since cataract surgery may further increase the risk for the development and progression of AMD. In the future, prospective multicenter studies with well-designed RCTs are required to confirm our findings.

## 1. Introduction

Cataract and age-related macular degeneration (AMD) are the two important causes of visual impairment and blindness in elderly people [1]. Both conditions are strongly age related, and each has a number of identified risk factors [2]. With rapidly aging populations and longer life expectancy, the WHO has estimated that the number of people with cataract blindness will increase to 40 million in 2025 [3]. Cataract surgery is the most frequent operation in ophthalmology and results in improved visual function [4]. Neovascular AMD is characterized by subretinal choroidal neovascularization and can be treated by intravitreal injection of anti-VEGFs [5]. Notably, AMD is also a common retinal comorbidity in patients undergoing cataract surgery, and surgeons should have additional concerns when patients are also known to have AMD [6].

Clarifying whether there are different risks between types of AMD after cataract surgery is very important from an informed consent perspective. However, the potential for cataract surgery to increase the risk of developing AMD is a long-standing clinical question, [7, 8] and the findings from epidemiological studies regarding these associations have been inconsistent [8–19]. Since then, several other studies investigating cataract surgery for AMD have been published [17, 20, 21]. Ho et al. [17] and Daien et al. [20] reported an increased risk of developing AMD following cataract surgery, whereas Park et al. suggested that the association between cataract surgery and AMD is uncertain in the current era of phacoemulsification [21]. There are several possible reasons for the inconsistent findings among studies. Differences in the duration of follow-up, response rates, sample source, study design, and strategies for adjusting for confounders could affect the results and might explain the inconsistent findings [7]. A previous meta-analysis of the association between cataract surgery and the risk of AMD was performed, but the possible influencing factors were not specifically evaluated [22, 23].

To address these issues, we conducted a meta-analysis to examine the association between cataract surgery and the development and progression of AMD, allowing for differences in the sample source, AMD type, and duration of follow-up.

## 2. Materials and Methods

### 2.1. Search Strategy

We attempted to conform to the Meta-analysis of Observational Studies (MOOSE) guidelines in the report of this meta-analysis [24]. This meta-analysis was registered at PROSPERO (CRD42017077962). Randomized controlled trials (RCTs) were included in this study in order to provide robust evidence with high-quality existing literature [25, 26]. Cohort studies and case-control studies were also included, which could address the temporal relationship between cataract surgery and AMD. We conducted a systematic literature search in August 2020, using Embase and PubMed, with the following search terms: cataract extraction (cataract extr^*∗*^, phaco^*∗*^), macular degeneration, choroidal neovascularization, and geographic atrophy. The detailed search strategy for the Embase and PubMed databases is described in the protocol presented in Additional file 1. Manual searches of all relevant trials and review articles were also performed. There were no language restrictions in the search for trials. We also reviewed all relevant trials included in previous systematic reviews and meta-analyses [22, 27].

### 2.2. Eligibility Criteria

The inclusion criteria were as follows: (1) studies evaluating the effect of cataract surgery on the development or progression of AMD; (2) cohort studies, case-control studies, or RCTs; and (3) studies with summary estimates, such as odds ratios (ORs) or hazards ratios (HRs) with 95% confidence intervals (CIs), reported or calculated in the article. We excluded studies if they were cross-sectional studies because they could not establish a causal association between cataract surgery and AMD.

### 2.3. Data Extraction and Quality Assessment

All data from eligible studies were independently extracted by two independent investigators (Hongxun Li and Xinheng Zhao). The results were compared, and disagreements were resolved by consensus. We extracted the following information: first author, publication year, study design, sample source, sample size, age range of the study participants, follow-up time, AMD diagnosis and grading criteria, summary estimates and the corresponding 95% CIs, and confounding factors adjusted for. We assessed the methodological quality of observational studies according to the Risk Of Bias In Non-randomized Studies of Interventions (ROBINS-I) tool [28]. The Cochrane Collaboration's tool for assessing the risk of bias was used for randomized trials [29].

### 2.4. Outcome Measures

The primary outcomes were as follows: the incidence of early AMD, defined by the presence of soft indistinct drusen or drusen associated with retinal pigment epithelial (RPE) depigmentation or increased retinal pigment at follow-up when none of these lesions was present at baseline and the incidence of late AMD, defined as the appearance of neovascular AMD or geographic atrophy at follow-up in eyes without either lesion at baseline. An eye was excluded from the incidence calculations for early or late AMD if either was already present at baseline. The secondary outcome was the progression of AMD, defined as an increase in maculopathy severity from baseline to the follow-up examination [30].

### 2.5. Statistical Analyses

Most studies used logistic regression models to adjust for confounders; therefore, we chose adjusted ORs as the measure of association between cataract surgery and AMD. For studies [17, 31] reporting only RRs or HRs, the ORs were used as estimates of the RRs or HRs because the incidence of AMD is extremely low in the general population. When studies with the same population or subpopulation were available, we considered the estimates from the most recent or most informative report. For studies [11, 15] *t* that reported only stratified results (e.g., by AMD subtype), we adopted the strategy recommended by Hamling et al. to calculate a combined OR [32]. We performed stratified analyses by the duration of follow-up, adjustment for early-stage AMD lesions, sample source, AMD type, and AMD subtype to provide robust evidence and investigate potential sources of between-study heterogeneity. We also performed a sensitivity analysis by both fixed-effects and random-effects models. Although both models obtained similar findings, the results from the random-effects models are presented here because of the different study designs, durations of follow-up, and strategies for adjusting for confounders that were involved in the original trials. Under these circumstances, the random-effects model approach was more conservative in medical decision-making contexts [33]. Statistical heterogeneity among studies was assessed with I^2^ and chi-squared tests. Heterogeneity was considered significant if *P* < 0.1 and considered high if the I^2^ value was above 75% [34, 35]. Publication bias was assessed by Egger's test [36]. Data were analyzed using the use of STATA version 12.0 (StataCorp LP, College Station, TX).

## 3. Results

### 3.1. Included Studies and Study Characteristics

**Figure 1:**
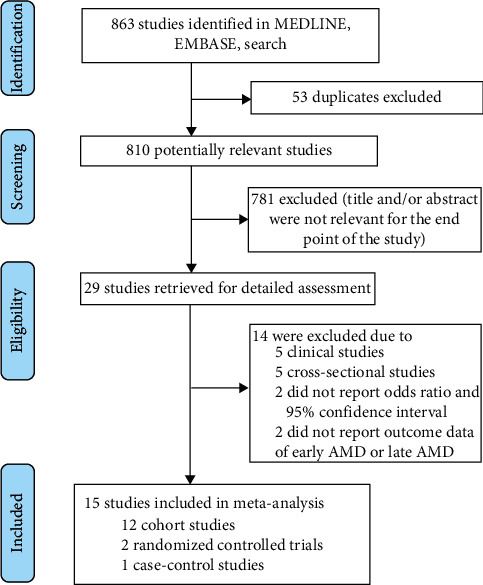
Study selection process. AMD = age-related macular degeneration.

We identified 863 studies from the initial literature search (Figure 1). After checking for duplicates and eliminating references that were deemed “not relevant” by titles and abstracts, we retrieved 29 full-text articles for detailed assessment. Our final analysis included 12 population-based cohort studies [9–12, 15–19, 30, 31, 37], 2 randomized controlled trials [25, 26], and 1 case-control study [8]. The baseline characteristics of the study participants are shown in Table 1. The studies were published between 1998 and 2017. The follow-up length of the cohort studies ranged from 1.5 to 20 years. Five studies [9, 10, 12, 30, 37] were from two cohort studies (the Beaver Dam Eye Study and the Blue Mountains Eye Study) with different follow-up times.  Table S1 shows the adjusted study-specific OR estimates (95% CIs) obtained from each study and the covariates that were adjusted for in the multivariate analyses (see Additional file 2). Seven studies (7 of 15) had a high risk of bias in at least one domain. The overall risk of bias of the included studies is shown in Additional file 3 (Figure S1. Quality of the included trials).

### 3.2. Quantitative Data Synthesis

**Figure 2:**
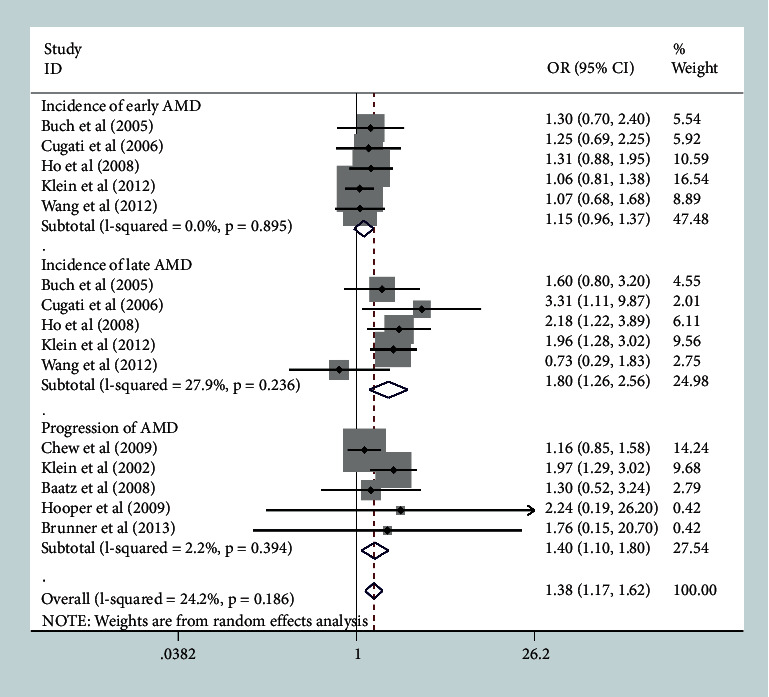
Risk estimates of the association between cataract surgery and the development and progression of age-related macular degeneration (AMD) in the overall analysis.

In the overall analysis, cataract surgery was significantly associated with a higher incidence of late AMD [10–12, 16, 18] (OR, 1.80; 95% CI, 1.26–2.56; *P* = 0.001, I^2^ = 27.9%), and a lower incidence of early AMD [10–12, 16, 18], but no statistically significant association was observed (OR, 1.15; 95% CI, 0.96–1.37; *P* = 0.139, I^2^ = 0%). We did find significant associations of cataract surgery with the progression of AMD (OR, 1.40; 95% CI, 1.10–1.80; *P* = 0.014, I^2^ = 2.2%) (Figure 2).  Table 2 shows the subgroup analyses based on the following stratified factors.

#### 3.2.1. AMD Subtype

**Figure 3:**
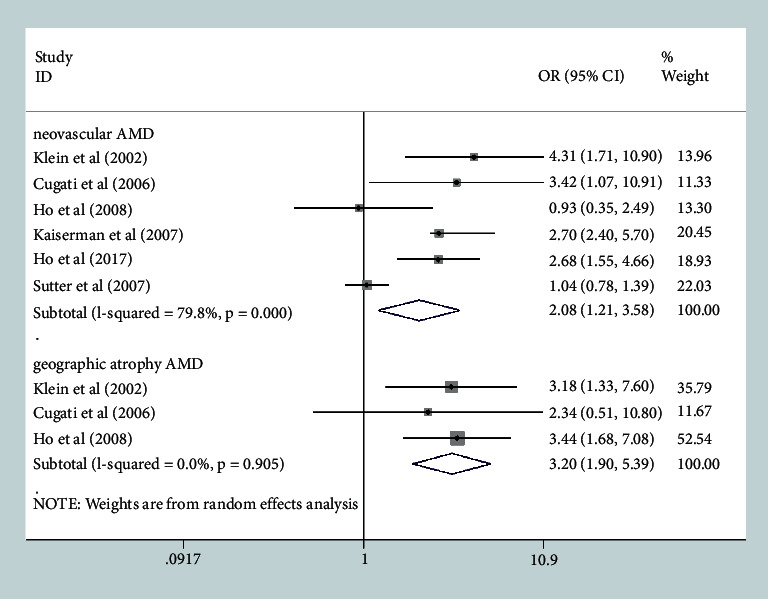
Forest plot evaluating the association between cataract surgery and the development of late age-related macular degeneration (AMD) based on AMD subtype.

When we stratified the studies by AMD subtype, the pooled OR for neovascular AMD was 2.08 (95% CI, 1.21–3.58; *P* = 0.008, I^2^ = 79.8%), and the heterogeneity was decreased substantially by removing clinic-based studies (OR, 2.57; 95% CI, 1.76–3.76; *P* ≤ 0.001, I^2^ = 28.8%) (Figure S2, see Additional file 4). The pooled OR for the geographic atrophy subtype of AMD was 3.20 (95% CI, 1.90–5.39; *P* ≤ 0.001, I^2^ = 0%) (Figure 3).

#### 3.2.2. Sample Source

In the analyses stratified by sample source, the pooled estimates for early AMD were systematically higher in population-based studies than in clinic-based studies, but the association was not statistically significant for either group (FigureS3, see Additional file 5); in contrast, the pooled estimates for late AMD were statistically significant for population-based studies (OR, 2.02; 95% CI, 1.50–2.72; *P* ≤ 0.001, I^2^ = 0%) (Figure S4, see Additional file 5).

#### 3.2.3. Adjusted Variables

Studies adjusted for early AMD lesions were considered. We found higher risk estimates for late AMD in the early AMD lesion-adjusted group than in the group that did not have early AMD lesions, but the difference was not statistically significant (Figure S5, see Additional file 7).

#### 3.2.4. Follow-Up Length

**Figure 4:**
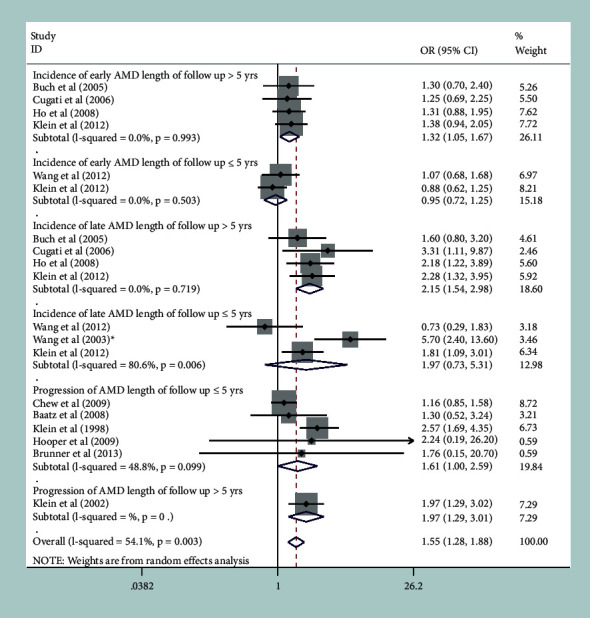
Forest plot evaluating the association between cataract surgery and the development and progression of age-related macular degeneration (AMD) based on follow-up duration. ^*∗*^The result from Blue Mountains Eye Study was included in this subgroup meta-analysis.

When we stratified the studies by follow-up duration, no statistically significant associations were observed between cataract surgery and the incidence of early AMD and late AMD in patients with a follow-up duration ≤5 years (Figure 4). In contrast, both ORs for longer follow-up durations (>5 years) showed significant associations. We also found significant associations of cataract surgery with the progression of AMD for a longer follow-up duration (OR, 1.97; 95% CI, 1.29–3.01; *P* = 0.002), but the pooled OR was not statistically significant for a shorter follow-up duration (Figure 4).

### 3.3. Sensitivity Analysis and Publication Bias

There was no evidence of publication bias for the incidence of AMD among studies by Egger's test (early AMD, *P* = 0.22; late AMD, *P* = 0.64). The sensitivity analyses showed that the OR and 95% CI were not altered substantially with either the fixed-effects model or the random-effects model (data not shown).

## 4. Discussion

Age-related eye diseases are expected to increase with an aging population. Cataract surgery is regarded as routine surgery and an effective treatment for cataract-induced vision loss. It remains a matter of debate whether cataract surgery itself increases the risk of AMD progression or AMD development [38]. Therefore, clarifying the importance of considering these risks will help both patients and physicians make more informed decisions.

In our meta-analysis, we found that cataract surgery was associated with an increased risk of late AMD development and AMD progression in the overall analyses. However, an association was not detected between cataract surgery and the incidence of early AMD in the overall analyses. A previous meta-analysis reported that the association between the progression of AMD and cataract surgery was not significant at the 6–12 month follow-up, and further research with a longer follow-up is encouraged [22]. Thus, when we compared the longer follow-up duration (>5 years) with the shorter follow-up duration (≤5 years), the odds were significantly different from each other. Our findings suggest that the greater the length of time since cataract surgery, the greater the odds were for the incidence of AMD and AMD progression.

To test the robustness of our results, we conducted subgroup analyses that permitted us to better understand the association in different subgroups. When we stratified the studies by AMD subtype, we found a stronger relationship with geographic atrophy than with neovascular AMD. The different pathophysiological characteristics in the natural history for both conditions differed by the same risk factor [39]. Our findings suggest that studies with primarily clinic-based patient samples have not found an association between cataract surgery and the incidence of late AMD. The possible reasons for the inconsistent findings could be differences in comorbidity distributions between clinic-based patients and population-based controls [7, 16]. Of note, the proportion of clinic-based patients with systemic comorbidities was higher than that of the healthy general population living in the community [40, 41]. Clinic-based patients who were included as controls in clinic-based studies may be more likely to have AMD [42–44]. This may be attributed to a selection bias in these studies. A better understanding of the reasons for the inconsistent findings across studies is important for clinical health practice and future research.

There are two possible reasons that might explain the association between cataract surgery and the development or progression of AMD. One possible explanation is light toxicity. Light, particularly short-wavelength light, can lead to the liberation of reactive oxygen species, which are highly toxic and can cause the formation of toxic lipid and protein peroxidation products [45]. Removal of the cataract exposes the retina to certain short wavelengths of light (e.g., ultraviolet or blue light), damaging the retina and increasing the risk of AMD development [39]. Additionally, the phototoxic effects in turn can lead to chronic inflammation by dendritic cells mediated by the local autoimmune response [46]. Most of the participants had surgery after the introduction of lenses with some blue-light-blocking ability. However, there is no evidence to date that supports the use of blue-blocking IOLs for the prevention of AMD [47]. A further possible explanation is the inflammation that could be induced during cataract surgery, especially in the early days of extracapsular/intracapsular cataract surgery.

However, studies also found that cataract surgery was beneficial for AMD patients in both improving visual acuity and improving quality of life [48, 49]. The majority of patients with late AMD (75.6%) still report significantly greater satisfaction with vision after cataract surgery, although the declining rate of visual acuity is faster in patients with AMD [50]. Physicians must balance the risks and benefits for patients with both visually significant cataracts and AMD [51]. Additionally, an informed discussion between physicians and patients is important based on currently available evidence. Our results show that the development and progression of AMD did not reach statistical significance for a follow-up duration less than 5 years postoperatively. Thus, cataract surgery seems to be beneficial for AMD patients when the age of AMD and cataract patients is taken into consideration.

The present meta-analysis had several limitations. First, the different strategies of the studies for adjusting for potential confounders limit the generalization of the results. We tried to overcome this problem by performing subgroup analyses in more homogeneous subsets of studies. Second, the classification of AMD severity differed among studies and could reduce the comparison of individual studies, but our meta-analysis showed low to moderate homogeneity among studies. Third, this meta-analysis was limited by the original study design of the trials and could not assess the efficacy of the type of implanted lenses and different surgical interventions for age-related cataracts in the development of AMD.

In conclusion, the results of the present meta-analysis suggest that cataract surgery may be a more important risk factor for those who are already at relatively high risk of incident late AMD. In addition, increasing the follow-up time since cataract surgery may further increase the risk for the development and progression of AMD. However, these findings should be considered carefully because some studies were rated as having a high risk of bias. In the future, prospective multicenter studies with well-designed RCTs are required to confirm our findings.

## Figures and Tables

**Table 1:** Characteristics of the included studies.

Authors (yr)	Region	Sample source	Sample size	Age (yrs)	Length of follow-up	AMD assessment	Overall risk of bias
*Cohort studies*							
Klein et al. (1998) [30]	US	Population-based	3684	43–86	5 yr follow-up	W	Moderate
Klein et al. (2002) [9]	US	Population-based	2764	43–86	10 yr follow-up	W	Moderate
Wang et al. (2003) [37]	Australia/USA	Population-based	6019	≥43	5 yr follow-up	W	Moderate
Buch et al. (2008) [19]	Denmark	Population-based	369	60–80	14 yr follow-up	W	Serious
Cugati et al. (2006) [10]	Australia	Population-based	3654	≥49	10 yr follow-up	W, I	Moderate
Kaiserman et al. (2007) [31]	Israel	Population-based	5913	＞50	1.5 yr follow-up	FP, FA	Serious
Ho et al. (2008) [11]	Netherlands	Population-based	6032	≥55	5.7 yr follow-up	I	Serious
Baatz et al. (2018) [20]	Germany	Clinic-based	1152	55–95	1 yr follow-up	FP, FA	Serious
Chew et al. (2009) [15]	USA	Clinic-based	4577	55–80	5 yr follow-up	A	Moderate
Klein et al. (2012) [12]	USA	Population-based	1913	43–86	20 yr follow-up	W	Moderate
Wang et al. (2012) [16]	Australia	Clinic-based	2029	≥65	3 yr follow-up	W	Moderate
Buch et al. (2009) [18]	Taiwan	Population-based	3465	50–74	5 yr follow-up	FP, FA	Serious

*Randomized controlled trials*							
Hooper et al. (2009) [25]	Australia	Clinic-based	56	71–92	6 mo follow-up	FA	Moderate
Brunner et al. (2013) [26]	Austria	Clinic-based	54	80	6 mo follow-up	FP	Serious
*Case-control studies*							
Sutter et al. (2007) [8]	Switzerland	Clinic-based	49	＞55	—	FP, FA	Serious

AMD = age-related macular degeneration; I = International AMD classification; W = Wisconsin AMD grading system; FP = fundus photography; FA = fluorescein angiography; A = Age-Related Eye Disease Study system classification.

**Table 2:** Subgroup analyses of cataract surgery and the incidence of age-related macular degeneration.

	Subgroups	No. of studies	Pooled OR	Significance			Heterogeneity
				95% CI	*P* value	*I* ^2^, (%)	*P* value
Study design	Cohort studies						
	Early AMD	5	1.15	0.96－1.37	0.139	0	0.895
	Late AMD	5	1.80	1.26－2.56	0.001	27.9	0.236
	Case-control studies						
	Late AMD	1	1.55	1.24－1.94	≤0.001	NA	NA
Follow-up length							
For early AMD	≤5 yrs	2	0.95	0.72－1.25	0.700	0	0.503
	＞5 yrs	4	1.32	1.05－1.67	0.018	0	0.993
For late AMD	≤5 yrs	3	1.97	0.73－5.31	0.181	80.6	0.006
	＞5 yrs	4	2.15	1.54－2.98	≤0.001	0	0.719
AMD type							
Sample source	Early sample source						
	Population-based	4	1.16	0.95－1.41	0.137	0	0.803
	Clinic-based	1	1.07	0.68－1.68	0.769	NA	NA
	Late						
	Population-based	4	2.02	1.50－2.72	≤0.001	0	0.728
	Clinic-based	1	0.73	0.29－1.83	0.503	NA	NA
Adjusted for early	Yes	2	2.39	1.43－3.99	0.001	0	0.626
AMD lesions	No	2	1.85	1.29－2.67	0.001	0	0.508

AMD = age-related macular degeneration; CI = confidence interval; OR = odds ratio.

## Supplementary Materials

Supplementary Materials Additional file 1: the detailed search strategy for Embase and PubMed database. Additional file 2: the adjusted study-specific OR estimates (95% CIs) obtained from each study and covariates that were adjusted for in the multivariate analyses. Additional file 3: the quality of the studies included in the meta-analysis. Additional file 4: forest plot evaluating the association between cataract surgery and the development of late AMD by removing clinic-based study for late AMD subtypes. Additional file 5: forest plot evaluating the association between cataract surgery and the development of early AMD based on sample source. Additional file 6: forest plot evaluating the association between cataract surgery and the development of late AMD based on sample source. Additional file 7: forest plot evaluating the association between cataract surgery and the development of late AMD based on adjustment for early AMD lesions.
