# Supplementary material for: Association between Cataract Surgery and Age-Related Macular Degeneration: A Systematic Review and Meta-Analysis
Source: J Ophthalmol. 2022 May 5;2022:6780901. doi: 10.1155/2022/6780901 (PMC9098349; doi:10.1155/2022/6780901)
Supplement: Supplementary Materials — Additional file 1: the detailed search strategy for Embase and PubMed database. Additional file 2: the adjusted study-specific OR estimates (95% CIs) obtained from each study and covariates that were adjusted for in the multivariate analyses. Additional file 3: the quality of the studies included in the meta-analysis. Additional file 4: forest plot evaluating the association between cataract surgery and the development of late AMD by removing clinic-based study for late AMD subtypes. Additional file 5: forest plot evaluating the association between cataract surgery and the development of early AMD based on sample source. Additional file 6: forest plot evaluating the association between cataract surgery and the development of late AMD based on sample source. Additional file 7: forest plot evaluating the association between cataract surgery and the development of late AMD based on adjustment for early AMD lesions. [file 6780901.f1.docx]

**Additional file 1**

**Formatted for PubMed search:**

1. “Macular Degeneration”[Mesh] OR “choroidal neovascularization”[Mesh] OR “geographic atrophy”[Mesh] OR “age-related maculopathy” OR “age related maculopathy” OR “age-related macular degeneration” OR “age related macular degeneration” OR “macular degeneration”

2. ‘‘Cataract extraction’’[Mesh] OR ‘‘Lens Implantation, Intraocular’’[Mesh] OR ‘‘Phacoemulsification’’[Mesh] OR‘‘Pseudophakia’’[Mesh] OR cataract extract* OR phakectom* OR OR cataract surger* OR lens implantation* OR intraocular lens* OR phaco* OR pseudophak* OR pseudo-phak* OR pseudofak* OR pseudo-fak*

3. “Cohort Studies”[Mesh] OR “longitudinal study”[Mesh] OR “prospective study”[Mesh] OR “Case-Control Studies” [Mesh] OR “Cohort Studies” OR “longitudinal study” OR “prospective study” OR “Case-Control Studies” OR (randomized controlled trial [pt] OR controlled clinical trial [pt] OR randomized [tiab] OR placebo [tiab] OR clinical trials as topic [mesh: noexp] OR randomly [tiab] OR trial [ti])

4. 1 AND 2 AND 3

**Formatted for Embase search:**

1. 'retina macula degeneration'/exp OR 'retina macula degeneration'

2. 'retina degeneration'/exp OR 'retina degeneration'
3. 'retina neovascularization'/exp OR 'retina neovascularization'

4. 'geographic atrophy'/exp OR 'geographic atrophy'

5. 'age related macular degeneration'/exp OR 'age related macular degeneration'

6. 'macular degeneration'/exp OR 'macular degeneration'
7. or/1-5

8. 'cataract extraction'/exp OR 'cataract extraction'
9. 'lens implantation'/exp OR 'lens implantation'
10. 'phacoemulsification'/exp OR phacoemulsification
11. 'pseudophakia'/exp OR pseudophakia
12. or/6-9

13. 'cohort analysis'/exp OR 'cohort analysis'

14. 'longitudinal study'/exp OR 'longitudinal study'

15. 'prospective study'/exp OR 'prospective study'

16. 'case control study'/exp OR 'case control study'

17. random* OR blind* OR placebo OR 'meta analysis'

18. or/13-17

19. 7 and 12and 18

**Additional file 2**

**Table S1. Estimates of the Association between Cataract Surgery and Age-related Macular Degeneration.**

**Phakia *vs*. nonphakic (Odd Ratio, 95% Confidence Interval)**

**Authors Early AMD Late AMD Neovascular AMD Geographic atrophy Factors Adjusted for in the Analyses**

**Cohort Studies**

Klein et al (1998) 1.73 (0.93-3.21) 2.80 (1.03-7.63) 1.67 (0.39-7.18) 3.49 (0.80-15.16) Age, sex, smoking, alcohol drinking status,

pulse pressure, hypertension, and vitamin use.

Klein et al (2002) 1.36 (0.82 – 2.23) 3.81 (1.89 – 7.69) 4.31 (1.71 – 10.9) 3.18 (1.33 – 7.60) Age, sex, smoking, heavy drinking status,

vitamin use, and systolic blood pressure.

Wang et al (2003) － 5.70 (2.40 – 13.6) 4.90 (1.90 – 12.4) 4.50 (1.4 – 14.7) Age, sex, smoking, and early-stage AMD

Buch et al (2005) 1.30 (0.70 – 2.40) 1.60 (0.80 – 3.20) － － Age, gender.

Cugati et al (2006) 1.25 (0.69 – 2.25) 3.31 (1.11 – 9.87) 3.42 (1.07 – 10.91) 2.34 (0.51 – 10.8) Age, sex, smoking, and early-stage AMD.

Kaiserman et al (2007) － － 2.70 (2.4 – 5.7) － Age, gender, country of birth, place of residency,

socioeconomic status, and chronic diseases status

Ho et al (2008) 1.31 (0.88 –1 .95) 2.18 (1.22 – 3.89) 0.93 (0.35 – 2.49) 3.44 (1.68 – 7.08) Age, sex, smoking, follow-up time, AMD stage.

Baatz et al (2008) － － 1.30 (0.52 – 3.24) － Age and baseline visual acuity.

Chew et al (2009) － 1.16 (0.85 – 1.58) 1.08 (0.65 – 1.77) 1.21 (0.82 – 1.80) Age, gender, race, smoking, AMD stage.

Klein et al (2012) 1.06 (0.81 – 1.38) 1.96 (1.28 – 3.02) － － Age, sex, smoking, education, drinking,

cardiovascular disease, diabetes, and diastolic

blood pressure.

Wang et al (2012) 1.07 (0.68 – 1.68) 0.73 (0.29 – 1.83) － － Age, sex, smoking, early AMD lesions.

Ho et al (2017) － － 2.68 (1.55 – 4.66) － Age, sex, geographical location,, income, diabetes,

hypertension, cardiovascular and hyperlipidaemia

**Randomized controlled trial**

Hooper et al (2009) － － 3.50 (0.34-35.9) － Age, sex, diabetes, hypertension.

Brunner et al (2013) － － 1.68 (0.14-19.9) － Age, gender.

**Case-control Studies**

Sutter et al (2007) － － 1.04 (0.78 – 1.39) － Paired-eye analysis.

**AMD = age-related macular degeneration.**

**Additional file 3**

**
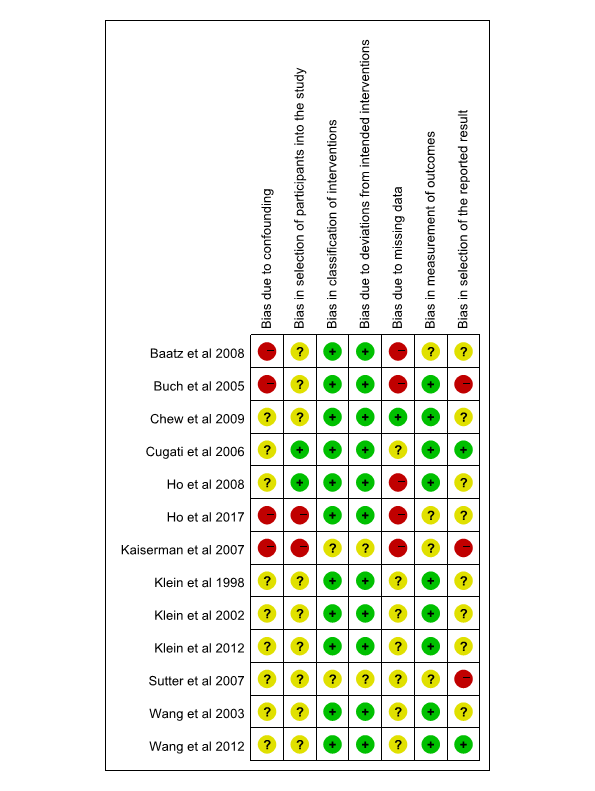
**
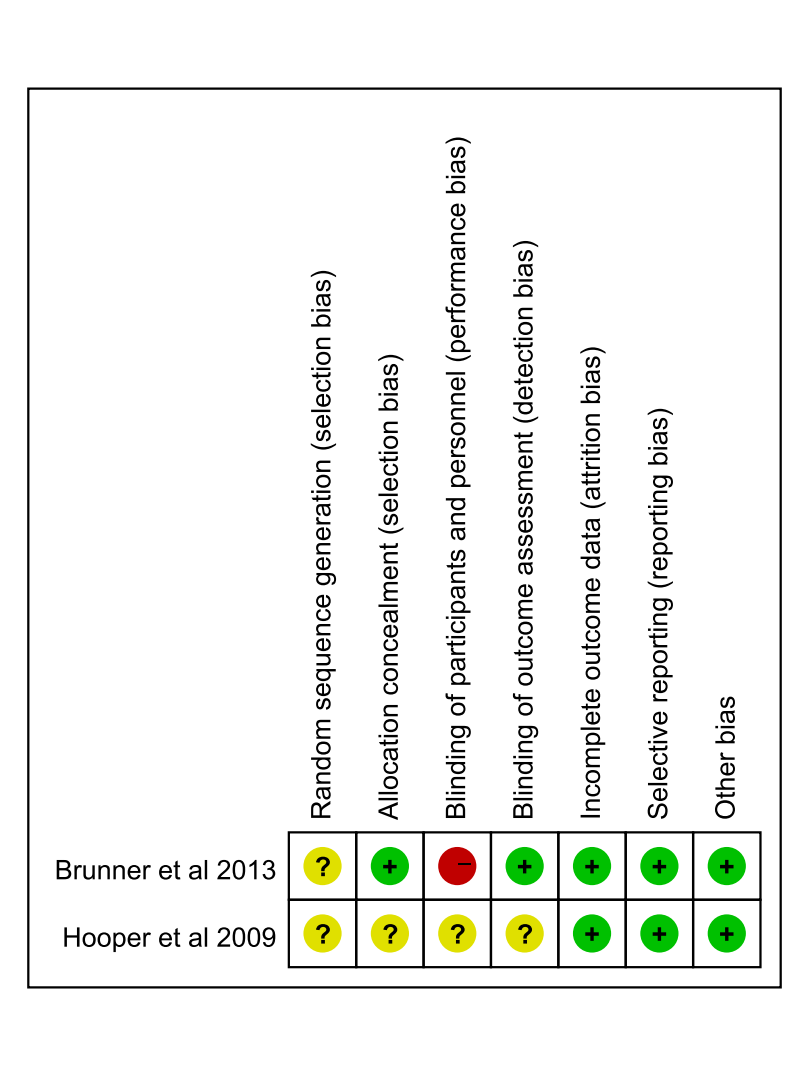


**Fig. S1.** Quality of the included trials. Green dot: “Low”, Yellow dot: “Moderate”, Red dot: “Serious”.

**Additional file 4**

**
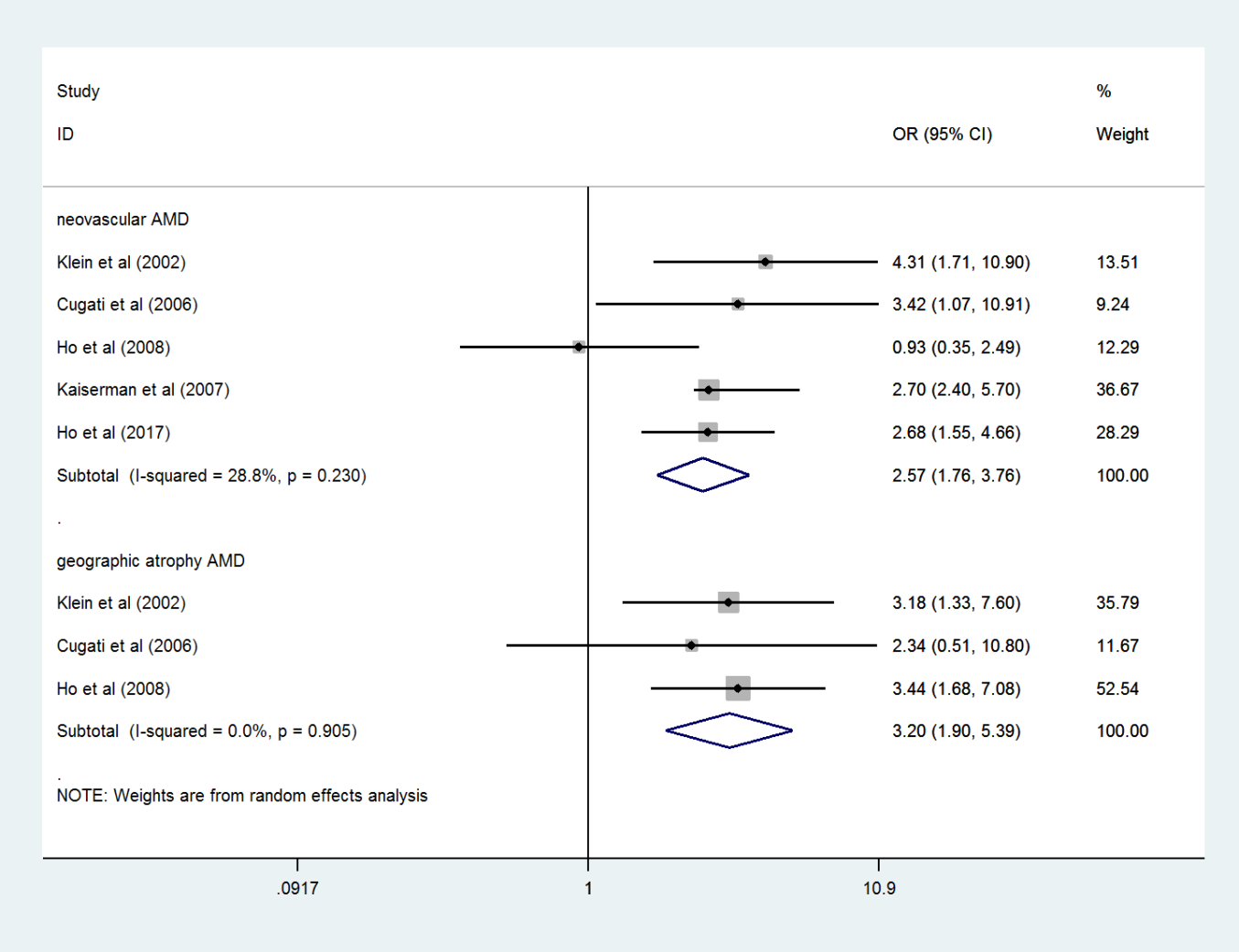
**

**Fig. S2.** Forest plot evaluating the association between cataract surgery and the development of late age-related macular degeneration (AMD) by removing clinic-based study for late AMD subtypes.

**Additional file 5**

**
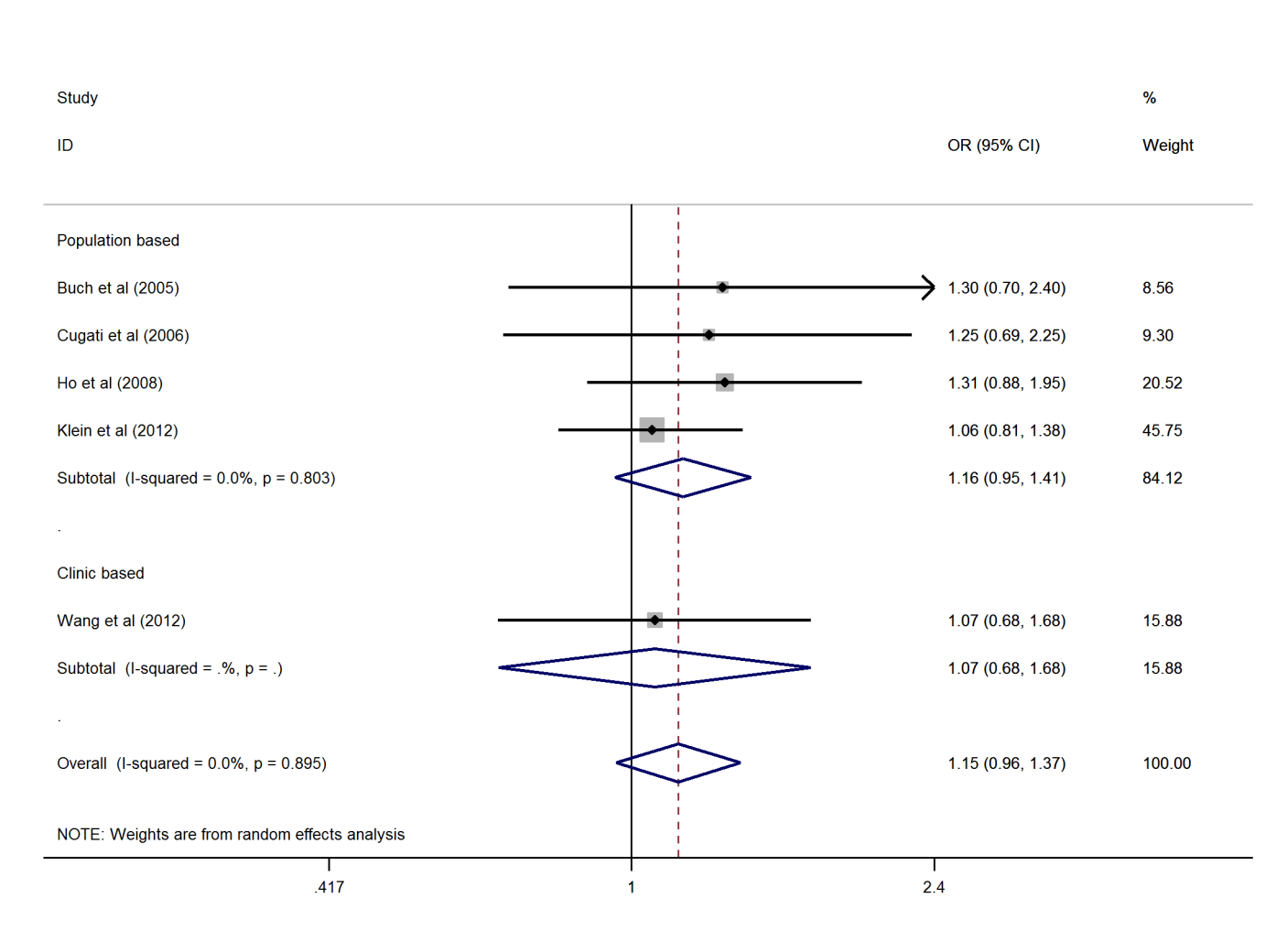
**

**Fig. S3.** Forest plot evaluating the association between cataract surgery and the development of early age-related macular degeneration (AMD) based on sample source.

**Additional file 6**

**
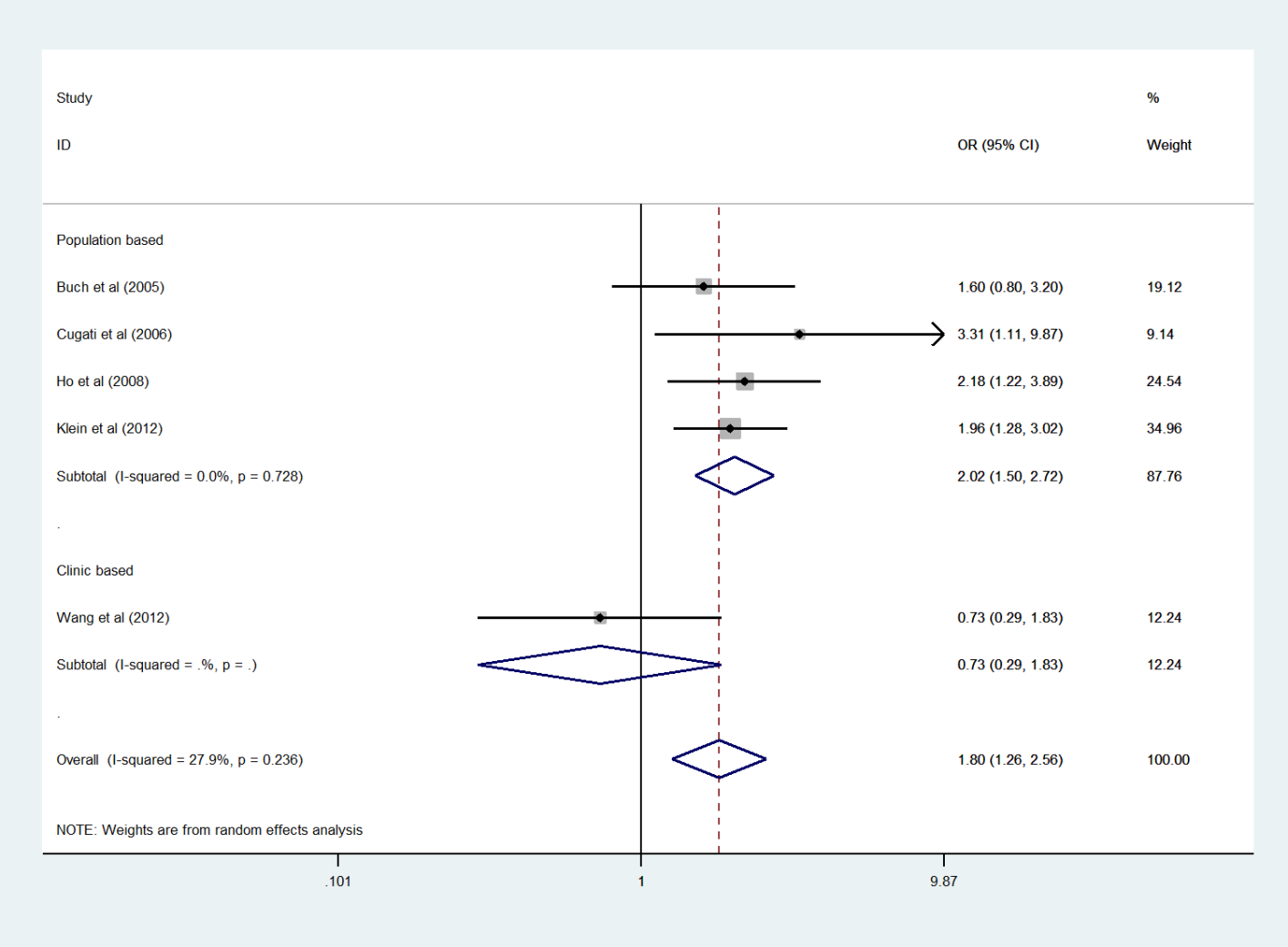
**

**Fig. S4.** Forest plot evaluating the association between cataract surgery and the development of late age-related macular degeneration (AMD) based on sample source.

**Additional file 7**

**
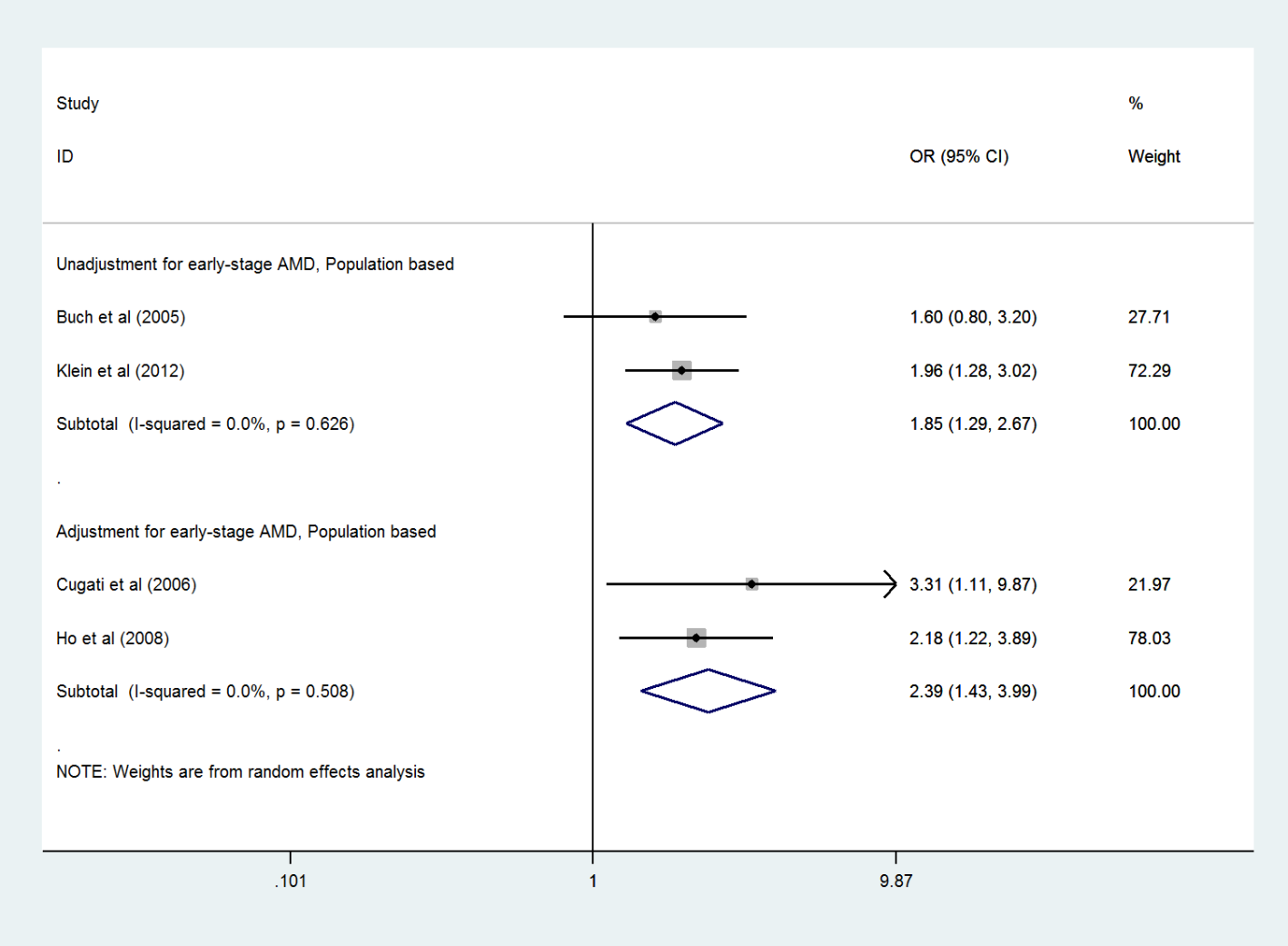
**

**Fig. S5.** Forest plot evaluating the association between cataract surgery and the development of late age-related macular degeneration (AMD) based on adjustment for early AMD lesions.
